# Too soon to grow: linking gut health to early puberty onset

**DOI:** 10.1038/s41390-025-04594-w

**Published:** 2025-11-15

**Authors:** Elizabeth Bell, Katheryn Bell, Heba M Ismail

**Affiliations:** Indiana University School of Medicine, Indianapolis, IN USA

Central precocious puberty (CPP) affects 1 in 5000–10,000 children and is 10 times more common in females than in males.^1^ If untreated, the accelerated epiphyseal growth that occurs during puberty could lead to short stature in adulthood due to premature epiphyseal closure as well as other potential psychosocial consequences.^1^ In this meta-analysis by Rodríguez Mazariegosa and group,^2^ the investigators explore the association between gut microbiome (GM) composition and CPP. They included both human and animal studies and identified specific bacterial signatures and microbial metabolites, such as short-chain fatty acids (SCFAs), that are associated with CPP in females and thus may influence activation of the hypothalamic-pituitary-gonadal (HPG) axis. While the findings suggest a potential link between GM alterations and early pubertal onset, causality remains unclear, highlighting the need for further studies.

The composition of the gut microbiome is influenced by multiple factors throughout life, with diet being the most significant.^3^ Other key influences include genetics, epigenetics, Body Mass Index (BMI), colon transit time, infections, antibiotic use, and environmental changes like immigration or long-term dietary shifts. While short-term stressors may cause temporary disruptions, prolonged or severe disturbances can result in dysbiosis—a harmful imbalance that impairs the gut’s beneficial functions and may contribute to disease.^3^

Obesity and a high BMI is one such factor that has been studied. For example, in a case-control study conducted by Liu et al., evidence suggested that obesity increased the risk of developing CPP. Obesity raised the likelihood of early puberty onset compared with normal-weight peers, with an adjusted odds ratio (aOR) of 1.78 (95% CI: 1.13, 3.48) for girls and 1.68 (1.09, 3.75) for boys. Furthermore, prolonged obesity, especially obesity lasting more than two years, was linked to an even higher risk of CPP_._^4^ Other factors, such as the use of antibiotics, which are known to alter the gut microbiome, and their influence on the timing of puberty have been explored. In a study conducted by Korpela et al. using statistical selection modelling, they identified the antibiotic type and timing most strongly linked to pubertal development for each sex. In girls, exposure to first- and second-generation cephalosporins before age 10 (measured by cumulative defined daily doses) was significantly associated with earlier pubertal progression (*p* = 0.004). In contrast, cephalosporin use showed no association with pubertal timing in boys (*p* = 0.97). For boys, the strongest—but still non-significant—association was a slight negative trend with macrolide use by age 10 (*p* = 0.12), while in girls, macrolide exposure was not related to puberty onset (*p* = 0.66). Additionally, a modest, non-significant positive trend was observed between BMI and pubertal timing in both sexes.^5^

Further confounding the findings is that there are sex specific differences that normally exist between male and female microbiota influenced by sex steroids, regardless of the timing of puberty. Several studies in humans and rodent models have shown that the fecal microbiomes of biological males and females are similar prior to puberty but then diverge in a sex-dependent manner during puberty.^6–11^ One of the earliest studies was performed at the cellular level in the 1980s, in which it was found that progesterone promoted the growth of *Bacteroides* species and *Prevotella intermedia*.^12^ A European study by Mueller et al. showed that healthy males had a higher abundance of Bacteroides-Prevotella than fertile females, while the microbiota of postmenopausal women did not differ from that of males.^13^ In the analysis by Korpela et al. described earlier, Clostridiales relative abundance increased, while Bacteroidales abundance and the Bacteroidales/Clostridiales ratio decreased with progression through puberty. These findings highlight potential microbiome contributions to sex-specific developmental trajectories during puberty.^5^

Moving beyond microbial composition, the mechanism by which the gut microbiome may influence puberty has been explored through the study of microbial function and metabolites. This meta-analysis expands on SCFAs,^2^ yet others such as bile acids or tryptophan metabolites have been considered. A study of postmenarcheal adolescents and older juvenile rats showed lower ratios of conjugated to deconjugated bile acids and higher levels of secondary bile acids, indicating developmental changes in bile acid metabolism. These shifts were accompanied by increased expression of the TGR5 receptor in the hypothalamus. TGR5 activation was shown to stimulate GnRH release via kisspeptin signaling, and overexpression of human TGR5 in the hypothalamus led to earlier puberty in female rats.^8^ The gut microbiome plays a significant role in tryptophan metabolism. Tryptophan and their metabolites can influence essential metabolic pathways, including phenylalanine and tyrosine biosynthesis and metabolism, the TCA cycle, glyoxylate and dicarboxylate metabolism, and tryptophan metabolism. A disruption in these pathways has been shown to correlate with elevated catecholamine derivatives as well as impaired serotonin synthesis, which can then collectively modulate GnRH secretion through different neuroendocrine pathways.^14,15^ Other mechanisms have been explored as well. Studies have shown that certain microbial species can secrete the neurotransmitters serotonin and dopamine as well as nitric oxide, which can then activate the HPG axis and directly stimulate pulsatile GnRH secretion.^15^ The gut microbiome, through its enzymes, can also play a role in the enterohepatic circulation of sex hormones. One such enzyme referenced in this meta-analysis,^2^ β-glucuronidase, breaks down estrogen and can potentially affect its bioavailability and impact on the body.

Lastly, the authors list limitations of their findings, including the inclusion of studies with only females, animal studies, human studies from a Chinese population, variability in how samples were collected and methods of analyses, thus limiting the generalizability of the findings, highlighting the need for broader studies that are more representative with standardized methods and that are longitudinal in nature.

In conclusion, while the meta-analysis confirmed associations between the gut microbiome composition and CPP based on data from several studies, the nature of this relationship remains unclear. Other studies have examined potential causation, yet more evidence from longitudinal studies in humans that account for the effect of other factors than can influence changes in the gut microbiome needs to be examined. Future studies aiming at interventions to alter the gut microbiome and, thereby, the CPP course in humans and animal models should also be considered and can aid in understanding causality.
